# Extra-abdominal Desmoid Tumor Mimicking Cervical Spine Schwannoma

**DOI:** 10.7759/cureus.3145

**Published:** 2018-08-14

**Authors:** Andrea Goldstein, Stanley Hoang, Douglas C Miller, Fassil B Mesfin

**Affiliations:** 1 Neurosurgery, University of Missouri, Columbia, USA; 2 Pathology, University of Missouri, Columbia, MO, USA

**Keywords:** extra-abdominal desmoid tumors, desmoid tumor, desmoid tumors, schwannoma, cervical spine schwannoma

## Abstract

Extra-abdominal desmoid tumors (DTs) are rare tumors of apparent fibroblastic origin with unpredictable clinical behavior. Though histologically benign and slow growing, DTs can be proliferative, aggressive tumors, invading the surrounding areas. DTs located extra-abdominally are most commonly found in the extremities or proximal structures like the shoulders, chest wall, and neck. Spinal involvement is very rare. Here, we describe a case where an extra-abdominal DT mimicked a schwannoma in the posterior cervical spine. A 67-year-old female patient presented with acute neck and bilateral shoulder pain. After attempting conservative treatments with no symptomatic relief, a magnetic resonance imaging of the cervical spine was obtained, showing a paraspinal mass in the posterior elements from C2 to C4. The computed tomography guided needle biopsy showed rare spindle cells, suggestive of a spindle cell neoplasm, and complete surgical resection was performed. The pathology report was consistent with fibromatosis, leading to a final diagnosis of the extra-abdominal desmoid. This case demonstrates a rare presentation of an unusual tumor that often manifests with nonspecific symptoms or no symptoms at all.

## Introduction

Extra-abdominal desmoid tumors (DTs) are rare tumors of apparent fibroblastic origin with unpredictable clinical behavior. Desmoid-type fibromatosis accounts for less than 3% of all soft tissue tumors and about 0.03% of all neoplasms [1-3], affecting two to four people per million, predominantly women [4-5]. Though histologically benign and slow growing, DTs can be proliferative, aggressive tumors, invading the surrounding areas. Recurrence rates are high, ranging from 24% to 77% depending on the patient’s age, location of the tumor, and resection margins [3].

Due to the high rate of invasion and local recurrence, as well as their variable natural history, DTs are difficult to treat clinically and can lead to significant morbidity and death. Prognoses are worse for tumors in the head and neck due to negative local consequences. Diagnosis and evaluation of the tumor is accomplished by using magnetic resonance imaging (MRI), while treatment for both intra- and extra-abdominal tumors requires a multidisciplinary approach [2-3]. Management includes nonsteroidal anti-inflammatory drugs (NSAIDs), anti-estrogen drugs, chemotherapeutic agents, radiation therapy, and surgical resection depending on the location and behavior of the tumor [2, 3, 6]. However, wide margin surgical resection is the current standard of care to prevent recurrence [2, 5].

Desmoids occurring extra-abdominally are most commonly found in the extremities or proximal structures like the shoulders, chest wall, and neck. Spontaneous formation is rare overall, but accounts for the majority of DTs [5]. As of 2015, only 14 cases of DTs localized to the spine have been reported, mostly occurring in the thoracic region [7]. Spinal DTs have also been observed in patients following posterior spinal instrumentation placement [4].

Interestingly, previous cases have identified a link between resection of schwannomas and subsequent development of extra-abdominal DTs [5, 8]. However, a patient with a DT mimicking a schwannoma in the posterior cervical spine has not been described to date.

## Case presentation

Case

**Figure 1:**
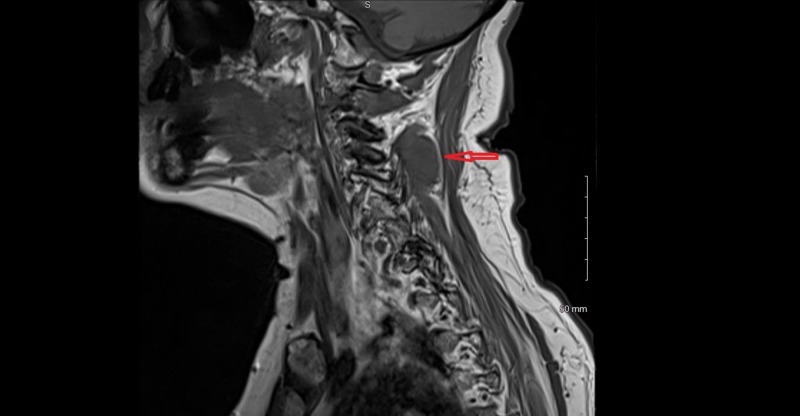
Parasagittal magnetic resonance imaging (MRI) of lesion. T1 weighted MRI of cervical spine with parasagittal image showing a hypointense mass in posterior elements from C2 to C4 measuring approximately 3.5 cm x 1.7 cm x 1.6 cm.

**Figure 2:**
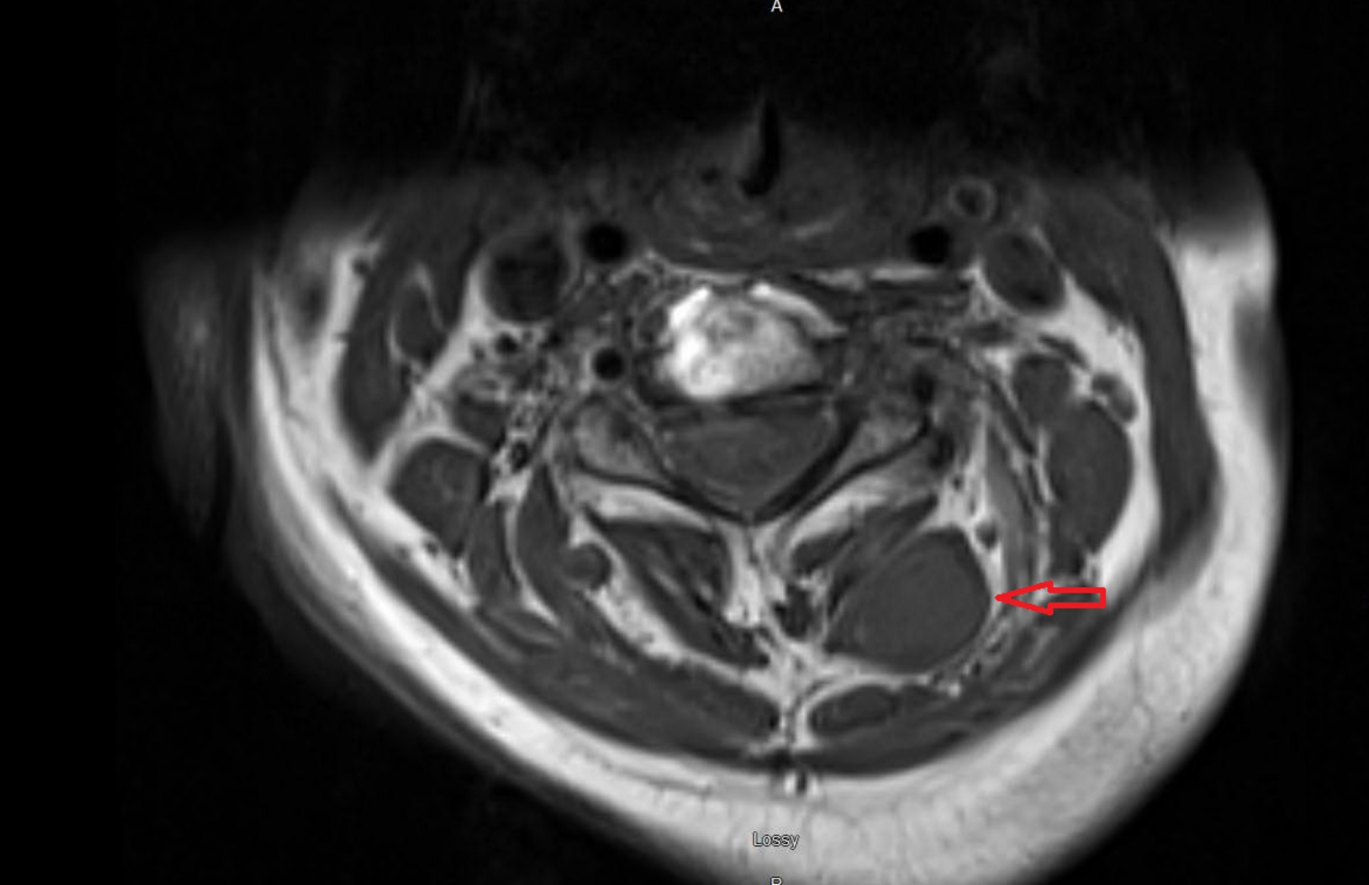
Axial MRI of lesion. T1 weighted MRI of cervical spine with axial images showing a hypointense mass in posterior elements from C2 to C4 measuring approximately 3.5 cm x 1.7 cm x 1.6 cm.

**Figure 3:**
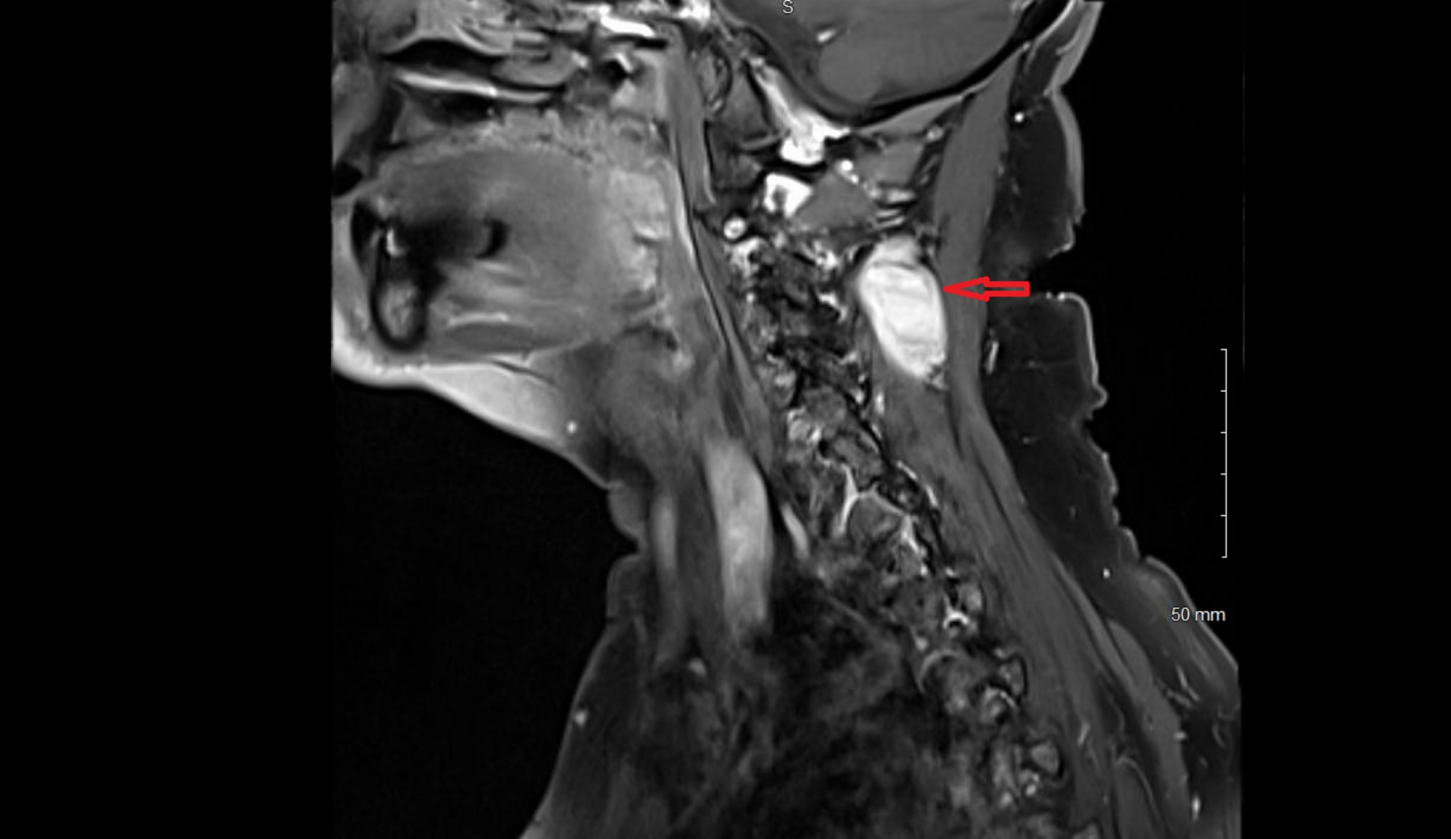
Parasagittal MRI postcontrast. Postcontrast MRI showing enhancement of the mass in the parasagittal plane.

**Figure 4:**
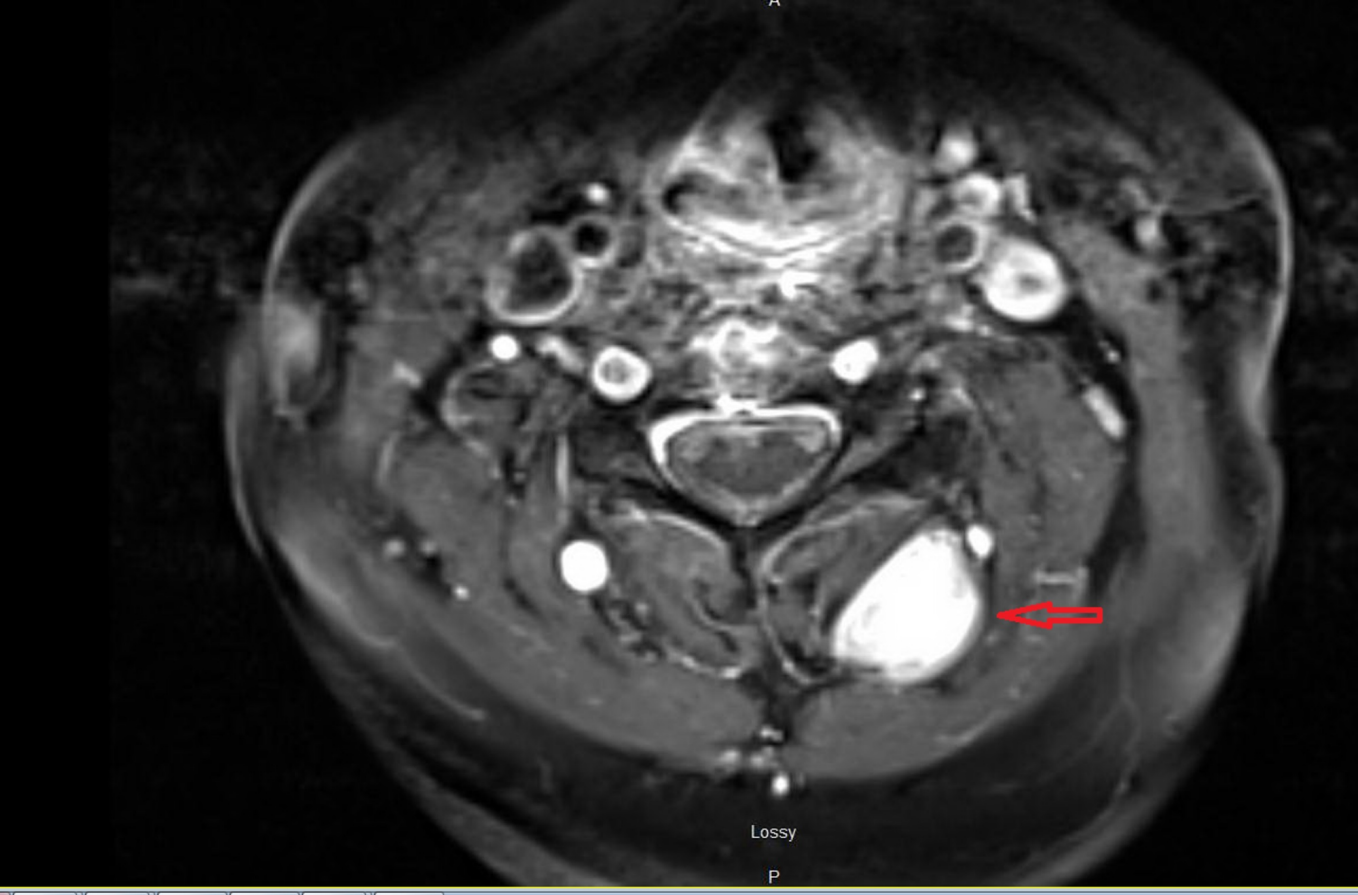
Axial MRI postcontrast. Postcontrast MRI showing enhancement of the mass in the axial plane.

A 67-year-old woman with a history of C5-C7 anterior cervical decompression and fusions presented with acute neck and bilateral shoulder pain. The patient did not have a history of trauma, significant family history, or syndromic findings suggestive of Gardner syndrome. Initially, she was managed conservatively using NSAIDs for pain management and physical therapy with minimal symptomatic relief. Her neurological exam, including motor, sensory and reflex testing was nonfocal. Due to the failure of conservative treatments, MRI of the cervical spine was obtained. This showed an approximately 3.5 cm x 1.7 cm x 1.6 cm paraspinal mass in the posterior elements from C2 to C4 (Figures 1-4); the mass was T2 hyperintense and homogenously enhanced (Figures 3-4). She underwent a computed tomography (CT) guided needle biopsy, which showed rare spindle cells, suggestive of a spindle cell neoplasm. Given the size of the mass and the intractable pain associated with it, surgical resection was performed.

Surgical observations

The mass was identified between spinal levels C2 and C4, below the muscular plane on the left side. The tumor was large, firm, and surrounded by muscle. The lesion was delineated from its attachment to the muscle in different planes. Direct stimulation of the lesion did not elicit an electromyographic response.

Postoperative care

**Figure 5:**
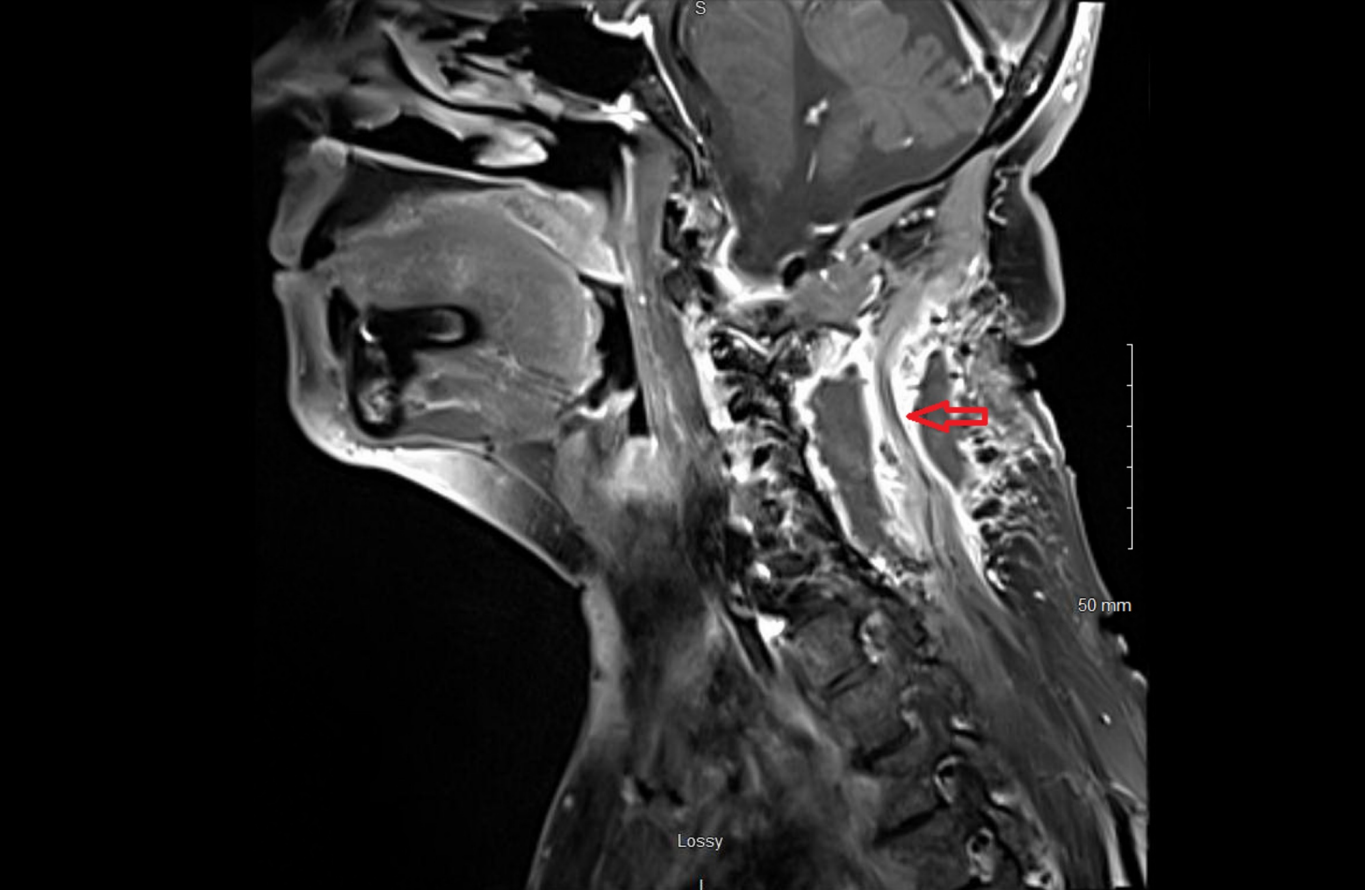
Parasagittal MRI postsurgical resection. T1 weighted enhanced MRI of the cervical spine four weeks after resection, showing gross total resection with associated postoperative changes in the parasagittal plane.

**Figure 6:**
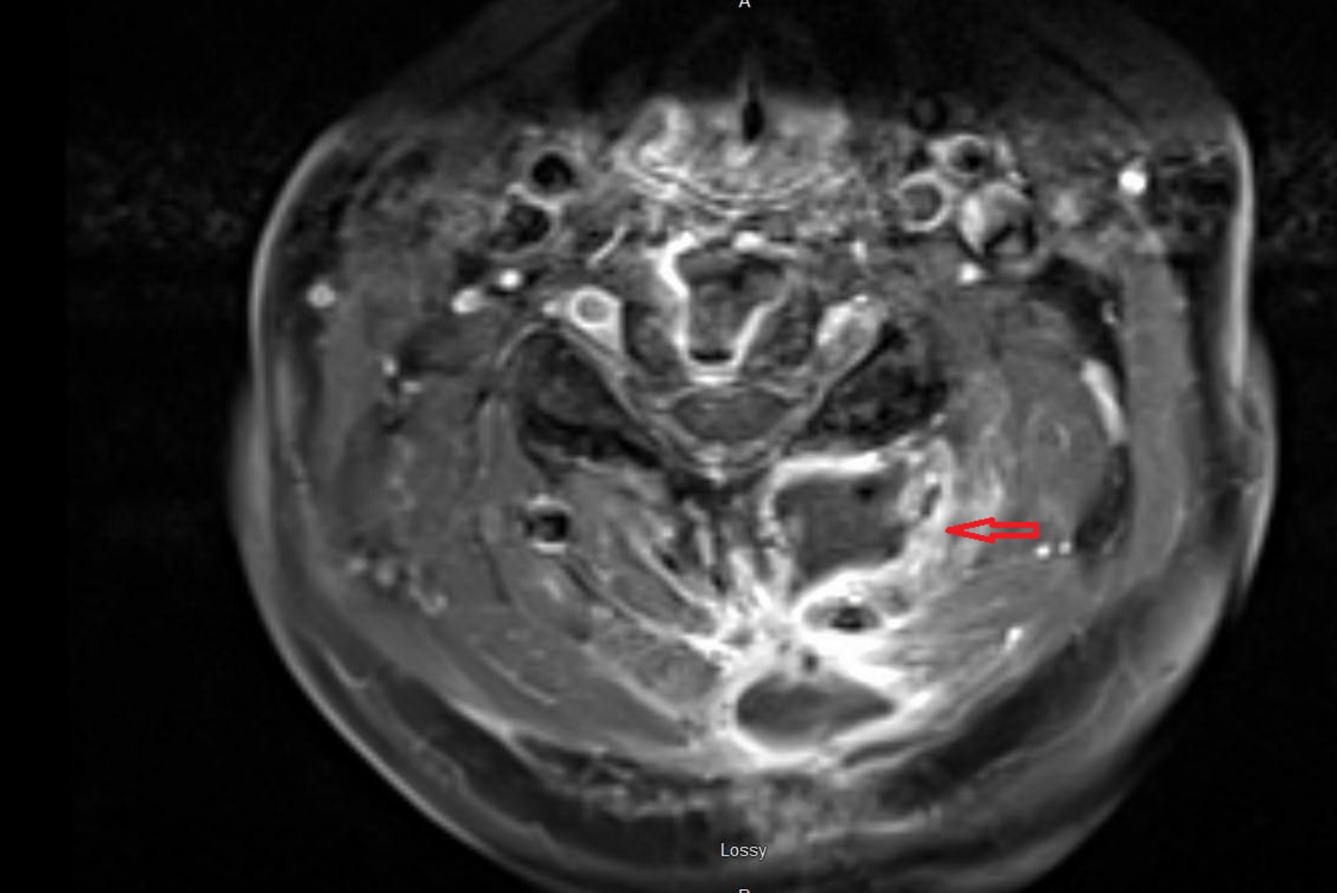
Axial MRI postsurgical resection. T1 weighted enhanced MRI of the cervical spine four weeks after resection, showing gross total resection with associated postoperative changes in the axial plane.

The patient had an uneventful postoperative course and was discharged home on postoperative day 2. A postoperative MRI confirmed gross total resection of the tumor (Figures 5-6). Given the complete resection and the ultimate pathological diagnosis (see below), oncology consultants advised that adjuvant therapy was unnecessary, but that close monitoring for recurrence was vital.

Pathology

**Figure 7:**
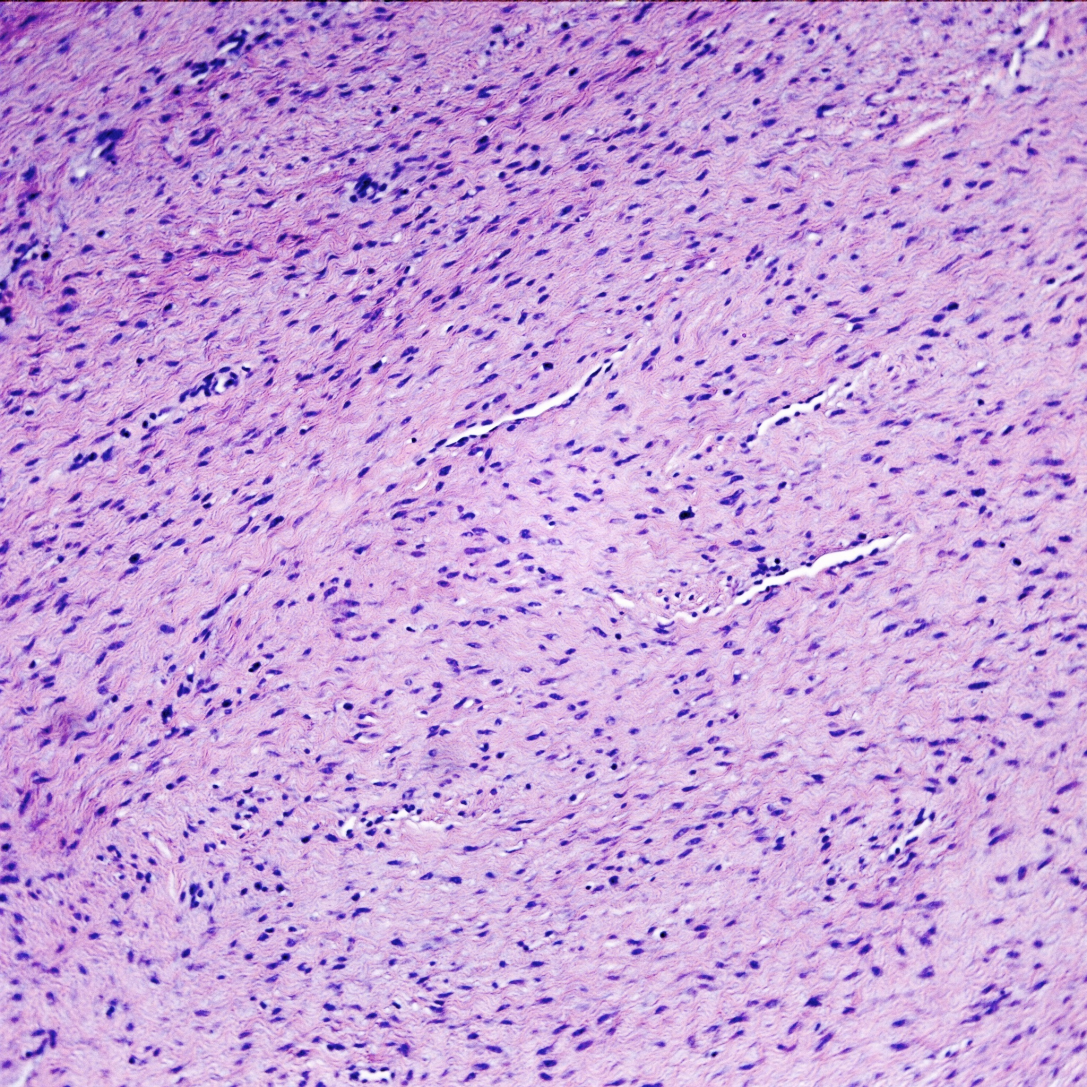
Hematoxylin and eosin (H&E) stain of lesion. The lesion is a spindle cell neoplasm infiltrating in a highly collagenous background. H&E stain, 100x.

**Figure 8:**
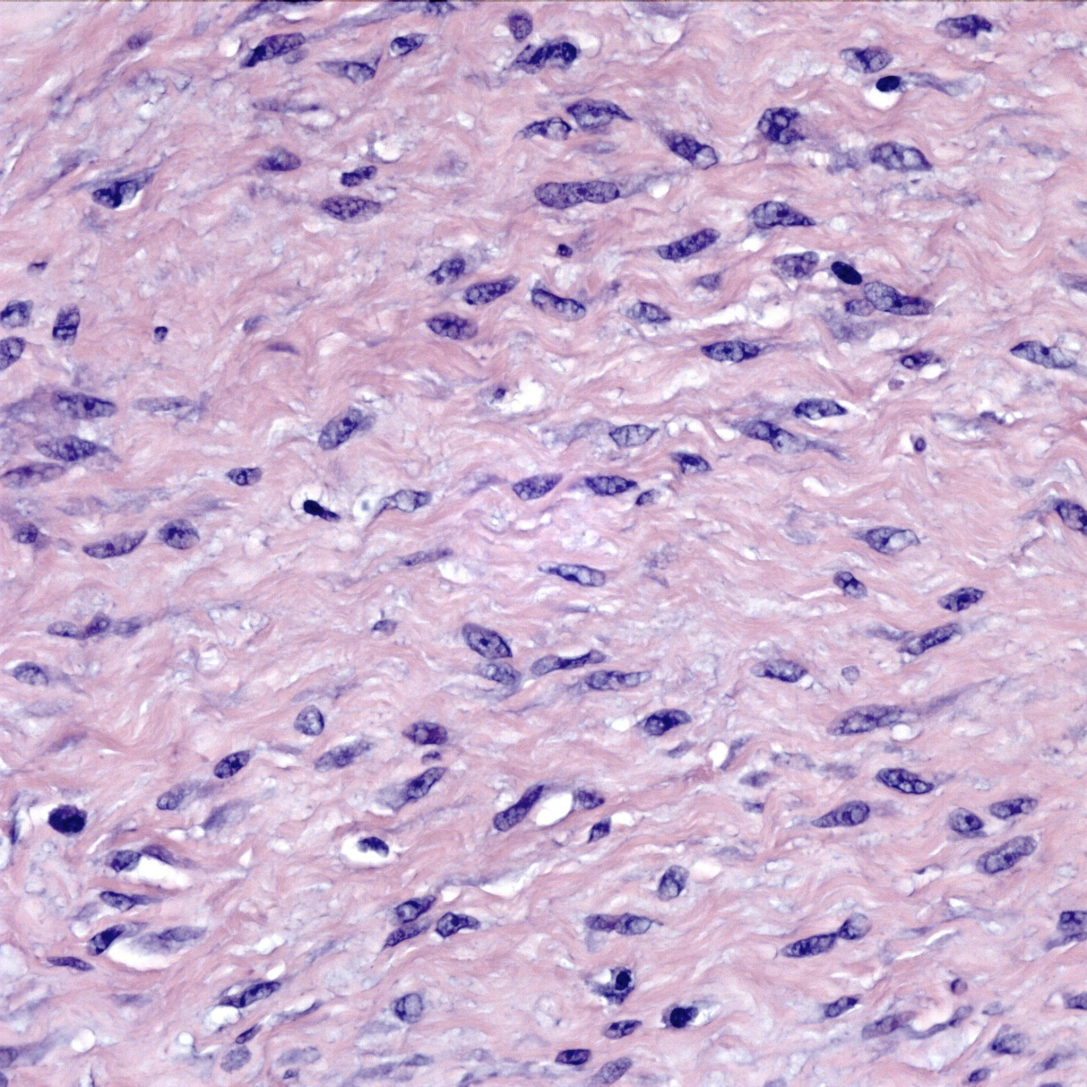
High power H&E stain. The nuclei are moderately pleiomorphic but mitotic figures are not evident. H&E stain, 400x.

**Figure 9:**
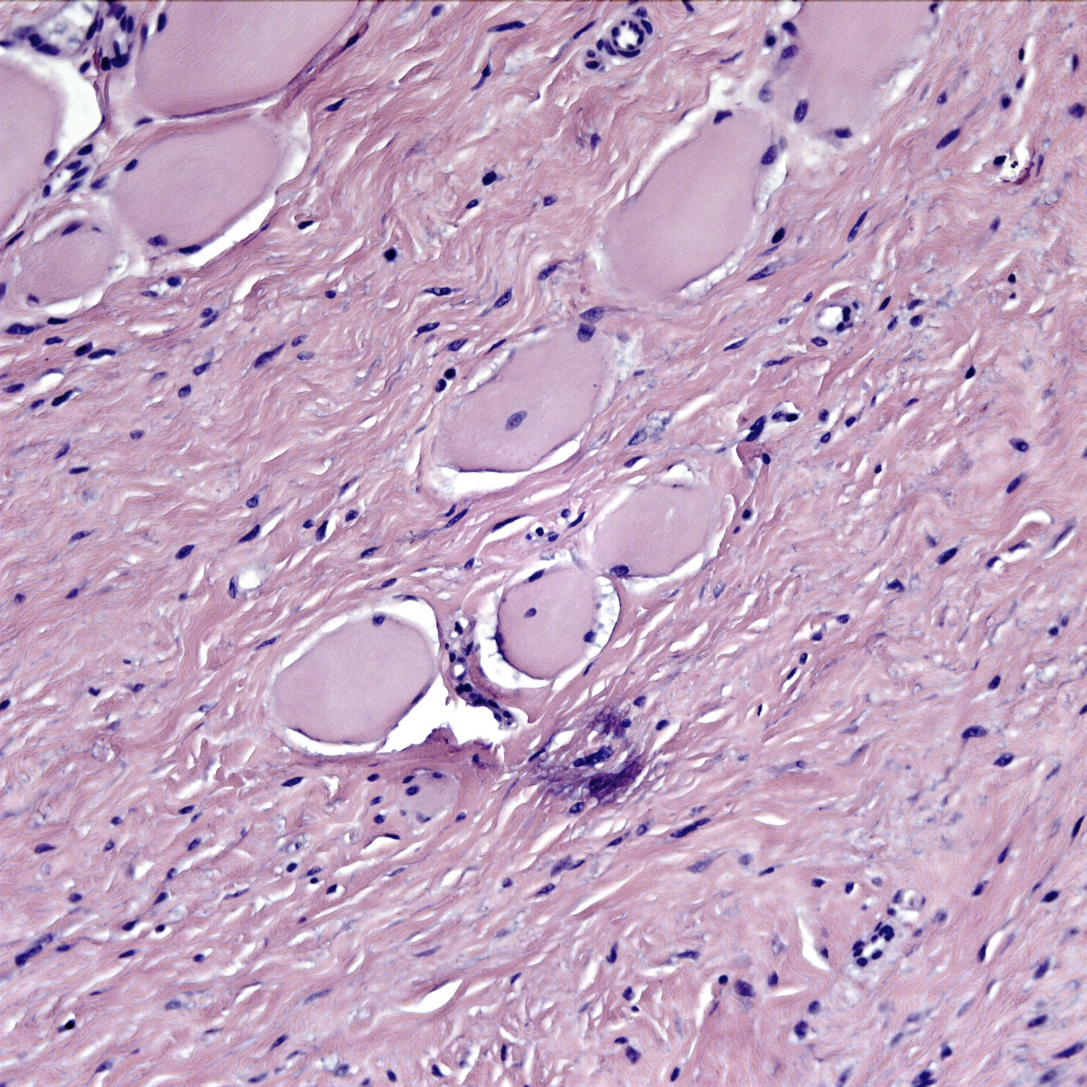
H&E stain showing infiltrative lesion. The lesion is infiltrative, with cells invading between and separating paraspinal skeletal muscle fibers. H&E stain, 200x.

**Figure 10:**
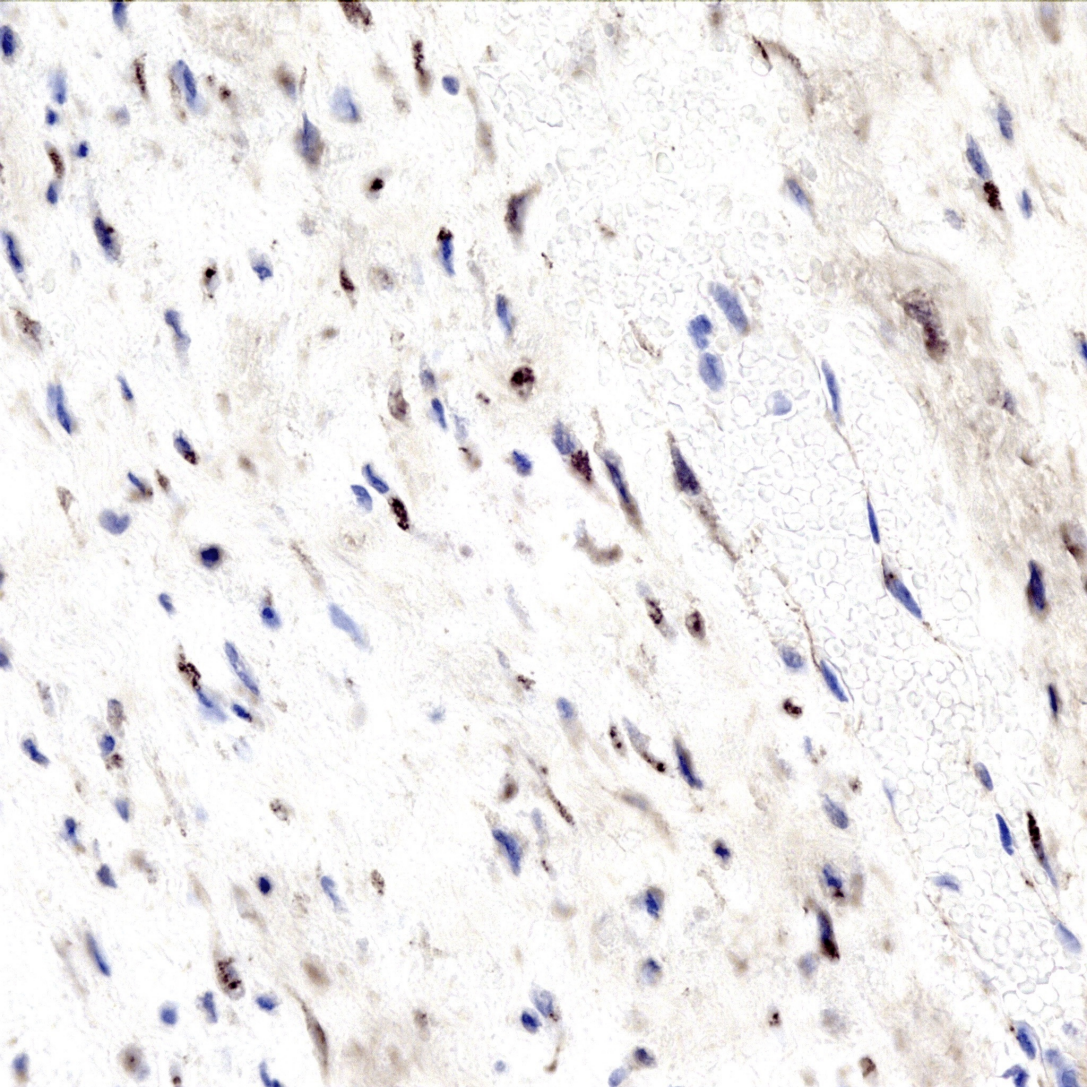
Beta-catenin immunostain. An immunostain for beta-catenin marks the cytoplasm of many of the spindle cells and there is also nuclear immunoreactivity in some of the lesional cells. 400x.

**Figure 11:**
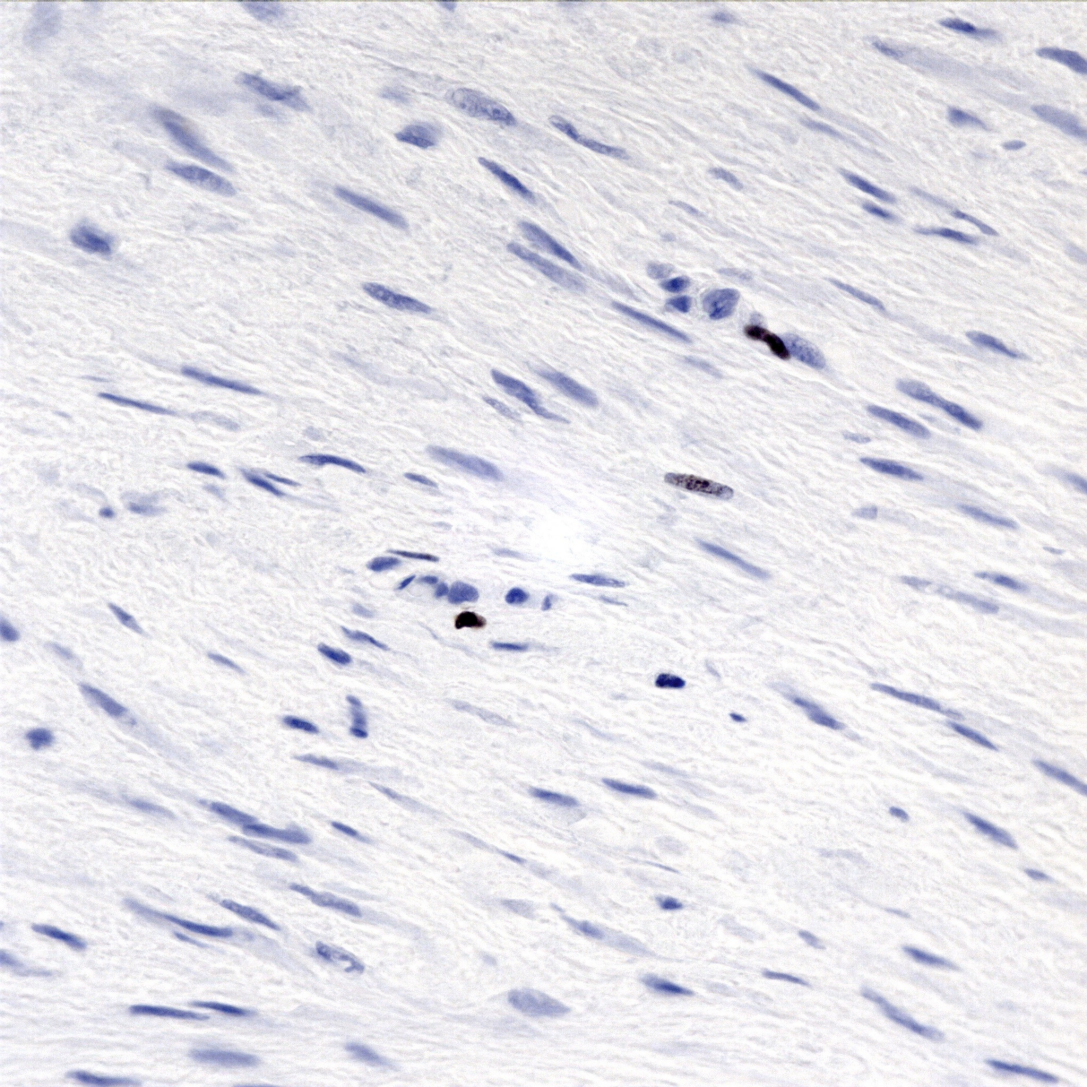
Ki67 immunostain. An immunostain for the cell cycle-associated antigen Ki67 marks the nuclei of well under 5% of the lesional cells. 400x.

The tumor was a spindle cell neoplasm of low to moderate cellularity without significant pleiomorphism (Figures 7-8). It infiltrated as single cells between skeletal muscle fibers entrapped in the collagenous matrix of the tumor (Figure 9). There was no necrosis or hypervascularity (a typical finding in extra-abdominal desmoids). The cells were not labeled by immunostains for S100 protein, smooth muscle actin, CD34, and e-cadherin, ruling out a neurofibroma, leiomyoma, primary endothelial tumor, and breast cancer metastasis, respectively. There was considerable cytoplasmic and some nuclear immunoreactivity for beta-catenin, a typical characteristic of fibromatoses/DTs (Figure 10), leading to a final diagnosis of extra-abdominal desmoid. Furthermore, a Ki67 immunostain demonstrated a low proliferative index of less than 5% (Figure 11).

## Discussion

Extra-abdominal DTs, or "aggressive fibromatoses" are locally aggressive, slow-growing neoplasms with no metastatic potential, but a substantial local recurrence rate. DTs typically infiltrate adjacent skeletal muscle cells and are otherwise within a densely collagenous or myxoid matrix, as seen in sections of the tumor from this patient.

The World Health Organization classifies DTs as intermediate or locally aggressive soft tissue tumors. This is to account for the absence of a demonstrable, metastatic potential combined with the high incidence of local recurrence, requi ring wide surgical margins to decrease the likelihood of recurrence [9]. Extra-abdominal DTs typically arise in the shoulder, chest wall, back, thigh, head, or neck [9]. Involvement of the cervical spine, as seen in this patient, is rare, especially in the absence of previous posterior spinal instrumentation placement.

The incidence of DTs changes depending on age and gender. In pediatric populations, most DTs are extra-abdominal and occur in both males and females equally [9]. The same gender distribution is seen in patients over 40 years of age, but the propensity for extra-abdominal lesions is not observed [9]. In this age group, the occurrence of intra- and extra-abdominal tumors is equivalent [9]. Patients are aged between puberty and 40 years and are typically females with intra-abdominal DTs. This case demonstrates a rare presentation of an unusual tumor in a patient who, as a 67-year-old woman, did not have a propensity for an extra-abdominal DT. 

Depending on the location of the tumor, extra-abdominal fibromatoses affect patients differently. DTs are often painless and most patients are asymptomatic. Patients seldom present with decreased joint mobility, neurological symptoms, or pain [9]. Interestingly, this patient’s chief concern was neck and shoulder pain, which could be a result of the lesion and nerve root impingement, or the lesion could be an incidental finding, unrelated to the pain.

As treatment of DTs requires an interdisciplinary approach, management strategies are determined individually for each patient.

## Conclusions

An extra-abdominal desmoid is a rare tumor. This case demonstrates a rare presentation of an unusual tumor that often manifests with nonspecific symptoms or no symptoms at all. Clinical suspicion and a tissue biopsy are required for diagnosis
